# The Prevalence of Plasmid-Mediated Colistin Resistance Gene *mcr-1* and Different Transferability and Fitness of *mcr-1*-Bearing IncX4 Plasmids in Escherichia coli from Pigeons

**DOI:** 10.1128/spectrum.03639-22

**Published:** 2023-02-28

**Authors:** Xiaoyu Lu, Wenhui Zhang, Mashkoor Mohsin, Mianzhi Wang, Jingui Li, Zhiqiang Wang, Ruichao Li

**Affiliations:** a Jiangsu Co-Innovation Center for Prevention and Control of Important Animal Infectious Diseases and Zoonoses, College of Veterinary Medicine, Yangzhou University, Yangzhou, Jiangsu, People’s Republic of China; b Institute of Comparative Medicine, Yangzhou University, Yangzhou, Jiangsu, People’s Republic of China; c Institute of Microbiology, University of Agriculture, Faisalabad, Pakistan; d Joint International Research Laboratory of Agriculture and Agri-Product Safety, the Ministry of Education of China, Yangzhou University, Yangzhou, Jiangsu, People’s Republic of China; University of Pittsburgh School of Medicine

**Keywords:** *Escherichia coli*, *mcr-1*, pigeons, IncX4, transferability, fitness

## Abstract

The prevalence of colistin-resistant bacteria limited the usage of colistin in the treatment of clinical multidrug-resistant Gram-negative bacterial infections. Here, we aimed to investigate the prevalence and molecular characterization of *mcr-1*-carrying isolates from pigeons close to humans following the ban on the use of colistin as an animal feed additive in China. Methods, including PCR, antimicrobial susceptibility testing, conjugation experiments, plasmid replicon typing, genome sequencing, bioinformatics analysis, measurement of growth curves, competition experiments, and plasmid stability assays were used to identify and characterize *mcr-1*-positive isolates. In total, 45 *mcr-1*-positive E. coli isolates were acquired from 100 fecal samples, and MICs of colistin ranged from 4 to 8 mg/L. The prevalence of *mcr-1*-positive E. coli isolates from pigeons was mainly mediated by IncX4 plasmids (39/45), including transferable *mcr-1*-bearing IncX4 plasmids with fitness advantage in 21 isolates, and nontransferable *mcr-1*-bearing IncX4 plasmids with fitness disadvantage in 18 isolates. There is a similar structure among the 6 *mcr-1*-bearing nontransferable IncX4 plasmids and 10 *mcr-1*-bearing transferable IncX4 plasmids in 16 E. coli isolates that have been sequenced. Plasmid transferability evaluation indicated that the same IncX4 plasmid has different transferability in different E. coli isolates. In conclusion, this study demonstrates that pigeons could act as potential reservoirs for the spread of *mcr-1*-positive E. coli in China. Transferability of IncX4 plasmids may be influenced by host chromosome in the same bacterial species. Additional research on the factors influencing the transferability of IncX4 plasmids in different bacterial hosts is required to help combat antimicrobial resistance.

**IMPORTANCE** The emergence of plasmid-mediated colistin resistance gene *mcr-1* incurs great concerns. Since the close proximity of pigeons with humans, it is significant to understand the prevalence and molecular characterization of *mcr-1*-positive isolates in pigeons, to provide a rationale for controlling its spread. Here, we found that the prevalence of *mcr-1*-positive E. coli from pigeons was mainly mediated by IncX4 plasmids. However, different transferability and fitness of *mcr-1*-bearing IncX4 plasmids in E. coli were observed, which demonstrated that transferability of IncX4 plasmids could be affected not only by genes on plasmids, but also by chromosomal factors in the same bacterial species. Our finding provided a new insight on studying the factors influencing the transferability of plasmids.

## INTRODUCTION

Colistin is applied as one of the last-resort therapies to treat carbapenem-resistant Enterobacteriales infections (1, 2). Due to the emergence of the plasmid-mediated mobile colistin resistance gene *mcr-1* in China and the rapid spread of *mcr-1*-positive isolates between humans and animals (3, 4), the Chinese government banned colistin as an animal growth promoter on 1 May 2017 (5). Withdrawal of colistin from animal feed may contribute to the decline of *mcr-1*-positive isolates, but they are still prevalent in humans, animals, and the environment, posing a serious threat to public health (6).

It is known that a large number of pigeons are living in close proximity to humans and animals all around the world. Numerous studies have indicated that pigeon feces is a possible reservoir spreading antibiotic-resistant bacteria (7 to 9). The prevalence of *mcr-1*-carrying isolates from pigeons likely contributes to its prevalence in the environment, humans, and animals. Therefore, it is significant to understand the prevalence characteristics of *mcr-1*-positive isolates in pigeons to provide a rationale for controlling its spread. Pigeon, harboring high nutritious value, is one important food animal in China (10). The prevalence of *mcr-1*-positive isolates from pigeons before 2017 in China has been reported (11), but the prevalence and genomic features of the *mcr-1*-carrying isolates were not fully explored after the ban of colistin as a feed additive. In this study, we aim to investigate the prevalence and molecular characteristics of *mcr-1*-bearing isolates from pigeons close to humans after China banned the use of colistin as an animal feed additive.

## RESULTS AND DISCUSSION

### Bacterial identification and resistance phenotypes.

A total of 45 *mcr-1*-bearing isolates were recovered from 100 collected fecal samples from pigeons in Jiangsu, China. All *mcr-1*-carrying isolates were identified as E. coli. This *mcr-1* prevalence of pigeon origin in Jiangsu (45/100, 45%) is much higher than previously reported in 2016 (11/46, 23.9%) (11). This indicated that there was no significant decrease in the prevalence of *mcr-1*-positive isolates from pigeons after China banned the use of colistin as an animal feed additive. Antimicrobial susceptibility testing revealed that MICs of colistin for all *mcr*-*1*-bearing isolates ranged from 4 to 8 mg/L (Table 1). These 45 isolates exhibited a high rate of resistance to doxycycline (66.67%, 30/45) and amoxicillin (64.44%, 29/45). In addition, partial isolates showed resistance to enrofloxacin (42.22%, 19/45), florfenicol (33.33%, 15/45), ceftiofur (28.89%, 13/45), and aztreonam (24.44%, 11/45). One isolate LP5-1 conferred resistance to meropenem with the MIC 16 mg/L (Table 1). Further PCR and Sanger sequencing proved that LP5-1 was a *bla*_NDM-5_-bearing isolate using primers described earlier (12).

**TABLE 1:** The MICs of 45 *mcr-1*-positive E. coli isolates against 10 antimicrobials^*a*^

Strain IDs	CST	FFC	CFF	DOX	ATM	STR	AMX	ENR	MEM	RIF
LP53-1	8	>64	>64	8	32	>64	>64	2	≤0.125	8
LP63-1	8	>64	1	>64	≤0.25	8	>64	1	≤0.125	8
LP23-1	4	>64	>64	16	16	>64	>64	2	≤0.125	8
LP15-1	8	>64	0.5	8	≤0.25	>64	4	≤0.25	≤0.125	8
LP21-1	8	>64	≤0.25	8	≤0.25	>64	4	0.5	≤0.125	8
LP98-1	8	>64	>64	8	16	>64	>64	0.5	≤0.125	4
LP12-1	8	>64	>64	16	8	>64	>64	1	≤0.125	8
LP45-1	8	4	≤0.25	16	≤0.25	8	4	≤0.25	≤0.125	8
LP82-1	8	8	>64	1	16	4	>64	1	≤0.125	4
LP37-1	8	4	0.5	16	0.5	16	4	0.5	≤0.125	8
LP20-1	8	4	≤0.25	2	≤0.25	8	>64	≤0.25	≤0.125	16
LP7-1	8	4	≤0.25	1	≤0.25	8	>64	≤0.25	≤0.125	4
LP41-1	8	2	≤0.25	16	≤0.25	8	4	0.5	≤0.125	8
LP4-1	8	2	≤0.25	1	≤0.25	4	>64	≤0.25	≤0.125	4
LP31-1	4	2	≤0.25	8	≤0.25	4	2	≤0.25	≤0.125	8
LP75-1	8	8	≤0.25	2	≤0.25	8	4	≤0.25	≤0.125	8
LP3-1	8	4	≤0.25	1	≤0.25	8	>64	≤0.25	≤0.125	4
LP8-1	4	8	≤0.25	32	≤0.25	>64	>64	1	≤0.125	>256
LP39-1	4	4	0.5	32	≤0.25	>64	>64	1	≤0.125	16
LP65-1	4	8	0.5	32	≤0.25	>64	>64	1	≤0.125	>256
LP81-1	4	8	0.5	16	≤0.25	>64	>64	1	≤0.125	>256
LP95-1	4	>64	1	64	≤0.25	8	>64	2	≤0.125	8
LP50-1	4	4	>64	32	64	16	>64	32	≤0.125	8
LP92-1	4	4	>64	32	32	32	>64	16	≤0.125	8
LP66-1	4	8	1	64	≤0.25	16	1	2	≤0.125	8
LP48-1	4	8	2	64	≤0.25	8	1	2	≤0.125	8
LP24-1	4	>64	0.5	32	≤0.25	16	>64	32	≤0.125	8
LP55-1	4	8	≤0.25	64	≤0.25	16	1	2	≤0.125	8
LP51-1	4	8	>64	32	32	>64	>64	16	≤0.125	8
LP91-1	8	4	>64	32	64	>64	>64	32	≤0.125	8
LP85-1	4	8	>64	32	32	>64	>64	16	≤0.125	8
LP84-1	4	4	0.5	4	≤0.25	8	8	≤0.25	≤0.125	16
LP10-1	4	4	0.5	4	≤0.25	8	4	≤0.25	≤0.125	16
LP49-2	4	>64	1	32	≤0.25	>64	>64	64	≤0.125	8
LP62-2	8	>64	2	32	≤0.25	>64	8	32	≤0.125	>256
LP43-1	4	>64	0.5	32	≤0.25	>64	>64	16	≤0.125	8
LP80-1	8	8	0.5	32	≤0.25	8	4	≤0.25	≤0.125	16
LP94-1	4	4	0.5	2	≤0.25	16	4	16	≤0.125	8
LP87-1	8	4	0.5	64	≤0.25	16	4	≤0.25	≤0.125	16
LP64-1	4	>64	≤0.25	32	≤0.25	>64	>64	1	≤0.125	>256
LP71-1	4	4	1	4	≤0.25	8	2	≤0.25	≤0.125	16
LP62-1	4	4	0.5	32	≤0.25	8	2	16	≤0.125	4
LP25-1	8	8	>64	32	16	16	>64	16	≤0.125	8
LP68-1	8	>64	>64	16	2	>64	>64	16	≤0.125	8
LP5-1	4	32	>64	64	≤0.25	16	>64	≤0.25	16	16
ATCC 25922	0.25	4	0.5	1	≤0.25	4	4	≤0.25	≤0.125	4

a CST, colistin; FFC, florfenicol; CFF, ceftiofur; DOX, doxycycline; ATM, aztreonam; STR, streptomycin; AMX, amoxicillin; ENR, enrofloxacin; MEM, meropenem; RIF, rifampicin.

### Transferability of *mcr-1* and *mcr-1*-associated plasmid types.

To investigate the transmissibility of the *mcr-1* gene, all *mcr-1*-harboring isolates were subjected to conjugation experiments. The genetic structures carrying the *mcr-1* gene of 23 E. coli isolates with colistin resistance phenotypes were successfully transferred into recipient E. coli C600 or J53. To learn the location of *mcr-1*, PCR-based replicon typing (PBRT) was performed for all 45 *mcr-1*-positive isolates and 23 transconjugants obtained by conjugation assays. Replicon types of 45 *mcr-1*-positive isolates and 23 transconjugants are shown in Table 2. We confirmed that *mcr-1* was located on IncX4-type plasmids in 39 isolates (Table 2) using specific primers IncX4-F and MCR1-RC-F targeting the IncX4-type plasmid replicon and the *mcr-1* gene with a product length of 2,854 bp (13). This indicated that *mcr-1*-bearing IncX4-type plasmid is the main vector for prevalence of *mcr-1* in pigeons from Jiangsu. It is well known that *mcr-1*-harboring IncX4-type plasmid is one of the most prevalent conjugative plasmids worldwide (13 to 15). However, *mcr-1*-bearing IncX4-type plasmids in 18 out of 39 isolates were nontransferable in this study. In the remaining 6 isolates carrying no *mcr-1*-harboring IncX4-type plasmid, we found that *mcr-1* is located on the IncI2 plasmids in two isolates, LP64-1 and LP71-1, by comparing the replicon types of parent isolates and their corresponding transconjugants (Table 2). The *mcr-1* in the other four isolates were nontransferable; therefore, we could not confirm the location of the *mcr-1* by PBRT (Table 2).

**TABLE 2:** Transferability of *mcr-1* and PBRT of 45 *mcr-1*-carrying isolates and corresponding transconjugants

Strain IDs	Replicon types	Recipient of conjugation	Transferability of *mcr-1*	Replicon types of transconjugants	Location of *mcr-1*
LP53-1	IncX4, IncFIB, IncF		Nontransferable		IncX4
LP63-1	IncX4, IncFIB, IncF		Nontransferable		IncX4
LP23-1	IncX4, IncFIB, IncF		Nontransferable		IncX4
LP15-1	IncX4, IncFIB, IncF		Nontransferable		IncX4
LP21-1	IncX4, IncFIB, IncF		Nontransferable		IncX4
LP98-1	IncX4, IncFIB, IncF		Nontransferable		IncX4
LP12-1	IncX4, IncFIB, IncF		Nontransferable		IncX4
LP45-1	IncX4, IncFIB, IncF		Nontransferable		IncX4
LP82-1	IncX4, IncFIB, IncF		Nontransferable		IncX4
LP37-1	IncX4, IncFIB, IncF		Nontransferable		IncX4
LP20-1	IncX4, IncFIB, IncF		Nontransferable		IncX4
LP7-1	IncX4, IncFIB, IncF		Nontransferable		IncX4
LP41-1	IncX4, IncFIB, IncF		Nontransferable		IncX4
LP4-1	IncX4, IncFIB, IncF		Nontransferable		IncX4
LP31-1	IncX4, IncFIB, IncF		Nontransferable		IncX4
LP75-1	IncX4, IncFIB, IncF		Nontransferable		IncX4
LP3-1	IncX4, IncFIB, IncF		Nontransferable		IncX4
LP8-1	IncX4, IncFIB, IncF, IncI1	J53	Transferable	IncX4	IncX4
LP39-1	IncX4, IncFIB, IncF, IncI1	C600	Transferable	IncX4	IncX4
LP65-1	IncX4, IncFIB, IncF, IncI1	J53	Transferable	IncX4	IncX4
LP81-1	IncX4, IncFIB, IncF, IncI1	J53	Transferable	IncX4	IncX4
LP95-1	IncX4, IncFIB, IncF, IncI1	C600	Transferable	IncX4	IncX4
LP50-1	IncX4, IncFIB, IncF, IncI2	J53	Transferable	IncX4	IncX4
LP92-1	IncX4, IncFIB, IncF, IncI2	J53	Transferable	IncX4	IncX4
LP66-1	IncX4, IncFIB, IncF, IncI2	J53	Transferable	IncX4	IncX4
LP48-1	IncX4, IncFIB, IncF, IncI2	J53	Transferable	IncX4	IncX4
LP24-1	IncX4, IncFIB, IncF, IncI2	C600	Transferable	IncX4	IncX4
LP55-1	IncX4, IncFIB, IncF, IncI2	C600	Transferable	IncX4	IncX4
LP51-1	IncX4, IncFIB, IncF, IncI1, IncI2	C600	Transferable	IncX4	IncX4
LP91-1	IncX4, IncFIB, IncF, IncI1, IncI2	C600	Transferable	IncX4	IncX4
LP85-1	IncX4, IncFIB, IncF, IncI1, IncI2	C600	Transferable	IncX4	IncX4
LP84-1	IncX4, IncI1, IncI2	C600	Transferable	IncX4	IncX4
LP10-1	IncX4, IncI1, IncI2	C600	Transferable	IncX4	IncX4
LP49-2	IncX4	C600	Transferable	IncX4	IncX4
LP62-2	IncX4, IncN	J53	Transferable	IncX4	IncX4
LP43-1	IncX4, IncI1	C600	Transferable	IncX4	IncX4
LP80-1	IncX4, IncFIB	J53	Transferable	IncX4	IncX4
LP94-1	IncX4, IncI2, IncI1, IncY	C600	Transferable	IncX4	IncX4
LP87-1	IncX4, IncFIB, IncI1		Nontransferable		IncX4
LP64-1	IncI2, IncI1	J53	Transferable	IncI2	IncI2
LP71-1	IncI2, IncI1, IncHI1, IncY	C600	Transferable	IncI2	IncI2
LP62-1	IncI2, IncFIB, IncF		Nontransferable		IncI2
LP25-1	IncI2, IncHI2, IncFIB, IncF		Nontransferable		IncI2
LP68-1	IncHI2, IncFIB, IncF		Nontransferable		IncHI2
LP5-1	IncFIB, IncF		Nontransferable		Chromosome

### Whole-genome sequencing analysis.

Twenty-one *mcr-1*-carrying isolates were selected to perform the genome sequencing, including 16 isolates carrying *mcr-1*-bearing IncX4 plasmids (based on their MICs, replicon typing, and transferability of IncX4 plasmids), 1 isolate carrying *mcr-1*-bearing IncI2 plasmid, and 4 isolates (LP25-1, LP62-1, LP68-1, and LP5-1) with undetermined location of *mcr-1* by conjugation and PBRT. By analyzing draft genomes, it was determined that the *mcr-1*-bearing contig also carried the IncHI2A replicon gene in isolate LP68-1, while the *mcr-1* gene and the IncI2 replicon gene were on the same contig in isolates LP25-1 and LP62-1. Therefore, we confirmed that isolates LP25-1 and LP62-1 both contained *mcr-1*-harboring IncI2 plasmids, and that LP68-1 carried an *mcr-1*-harboring IncHI2 plasmid. However, we are still unable to determine the localization of *mcr-1* in LP5-1 based on the draft genome.

**FIG 1:**
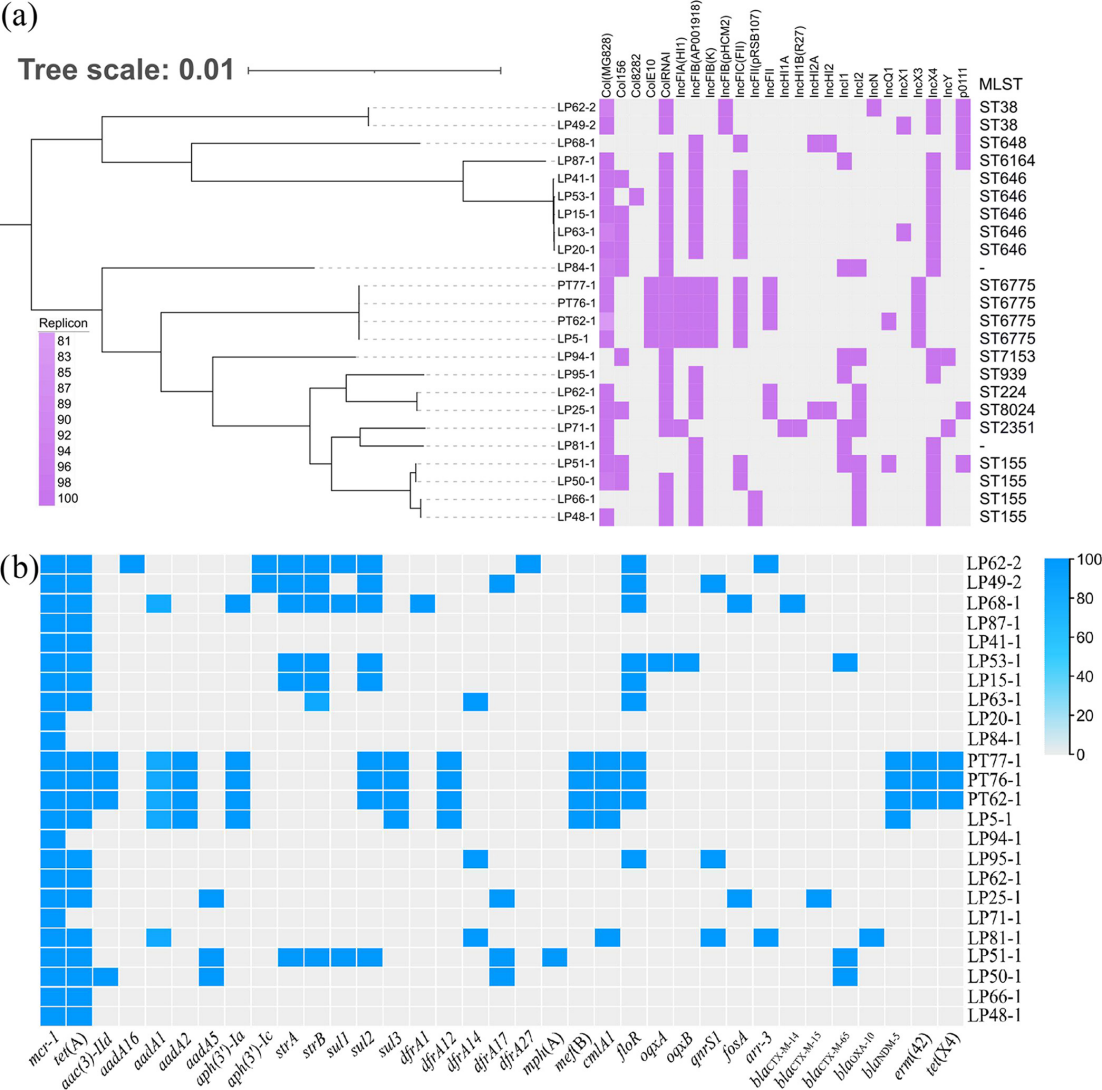
The genetic relationship of 21 *mcr-1*-carrying isolates from pigeons and the distribution of replicons and antimicrobial resistance genes. (a) Phylogenetic tree of 21 *mcr-1*-positive E. coli isolates and the distribution of replicons. The presence or lack of replicons is colored in purple or light gray, respectively. The legend shows the similarity (%) of the replicons. (b) The distribution of antimicrobial resistance genes. The blue rectangles indicate the presence of resistance genes. The legend indicates the similarity (%) of the antimicrobial resistance genes. For interpretation of the references to color in this figure legend, the reader is referred to the web version of this article. Three isolates (PT62, PT76 and PT77) co-harboring *mcr-1*, *tet*(X4) and *bla*_NDM-1_, that we had reported previously, were presented in this figure.

The phylogenetic tree showed that 21 E. coli isolates were grouped into two major clusters and were diverse. These distinct 21 *mcr-1*-positive isolates were assigned into 11 known sequence types (STs) (Fig. 1a), including ST646 (*n* = 5), ST155 (*n* = 4), ST38 (*n* = 2), ST224 (*n* = 1), ST8024 (*n* = 1), ST7153 (*n* = 1), ST939 (*n* = 1), ST648 (*n* = 1), ST2351 (*n* = 1), ST6164 (*n* = 1), and ST6775 (*n* = 1). Isolates LP81-1 and LP84-1 belonged to novel STs (Fig. 1a). Three isolates (PT62, PT76, and PT77) coharboring *mcr-1*, *tet*(X4), and *bla*_NDM-1_ were also acquired from this pigeon farm, and we had reported this previously (16). Isolate LP5-1 was very closely related to these three isolates and belonged to ST6775 (Fig. 1a). Han et al. reported that *mcr* genes were present among *fosA3*-carrying E. coli at the proportion of 77.8%. These *fosA3*-carrying E. coli were also isolated from pigeons but from Guangdong, China in 2016 (17). These *mcr*-bearing isolates in their study were categorized into the highly abundant STs that differed from the STs in our study, except for ST155. The phylogenetic relationship and multiple STs suggest that *mcr-1*-carrying isolates in pigeons from China are diverse.

### Prevalence of acquired antibiotic resistance genes.

Thirty-three resistance genes, conferring resistance to diverse antibiotics, were identified in these 21 isolates that have been sequenced. LP68-1, containing 12 resistance genes, was the isolate with the largest number of resistance genes among 21 isolates. With the exception of *mcr-1*, the tetracycline resistance gene *tet*(A) was the most prevalent and was present in 17 isolates. A total of nine genes conferring resistance to aminoglycoside were detected, including *aac(3)-IId* (*n* = 1), *aadA16* (*n* = 1), *aadA1* (*n* = 3), *aadA2* (*n* = 1), *aadA5* (*n* = 3), *aph(3′)-Ia* (*n* = 2), *aph(3′)-Ic* (*n* = 2), *strA* (*n* = 6), and *strB* (*n* = 7). Additionally, three sulfonamides resistance genes, *sul1* (*n* = 3), *sul2* (*n* = 6), and *sul3* (*n* = 1), and five trimethoprim resistance genes, *dfrA1* (*n* = 1), *dfrA12* (*n* = 1), *dfrA14* (*n* = 3), *dfrA17* (*n* = 4), and *dfrA27* (*n* = 1), were identified. Furthermore, macrolide resistance genes *mph*(A) (*n* = 1) and *mef*(B) (*n* = 1), phenicol resistance genes *cmlA1* (*n* = 2) and *floR* (*n* = 7), quinolone resistance genes *oqxA* (*n* = 1), *oqxB* (*n* = 1), and *qnrS1* (*n* = 3), fosfomycin resistance gene *fosA* (*n* = 1), and rifampicin resistance gene *arr-3* (*n* = 2) were found. Importantly, CTX-M-type extended-spectrum-β-lactamase-encoding genes *bla*_CTX-M-14_ (*n* = 1), *bla*_CTX-M-15_ (*n* = 1), and *bla*_CTX-M-65_ (*n* = 3), OXA-type extended-spectrum-β-lactamase-encoding gene *bla*_OXA-10_ (*n* = 1), and carbapenems resistance gene *bla*_NDM-5_ (*n* = 1) were also found (Fig. 1b).

### Genetic environments of *mcr-1*.

**FIG 2:**
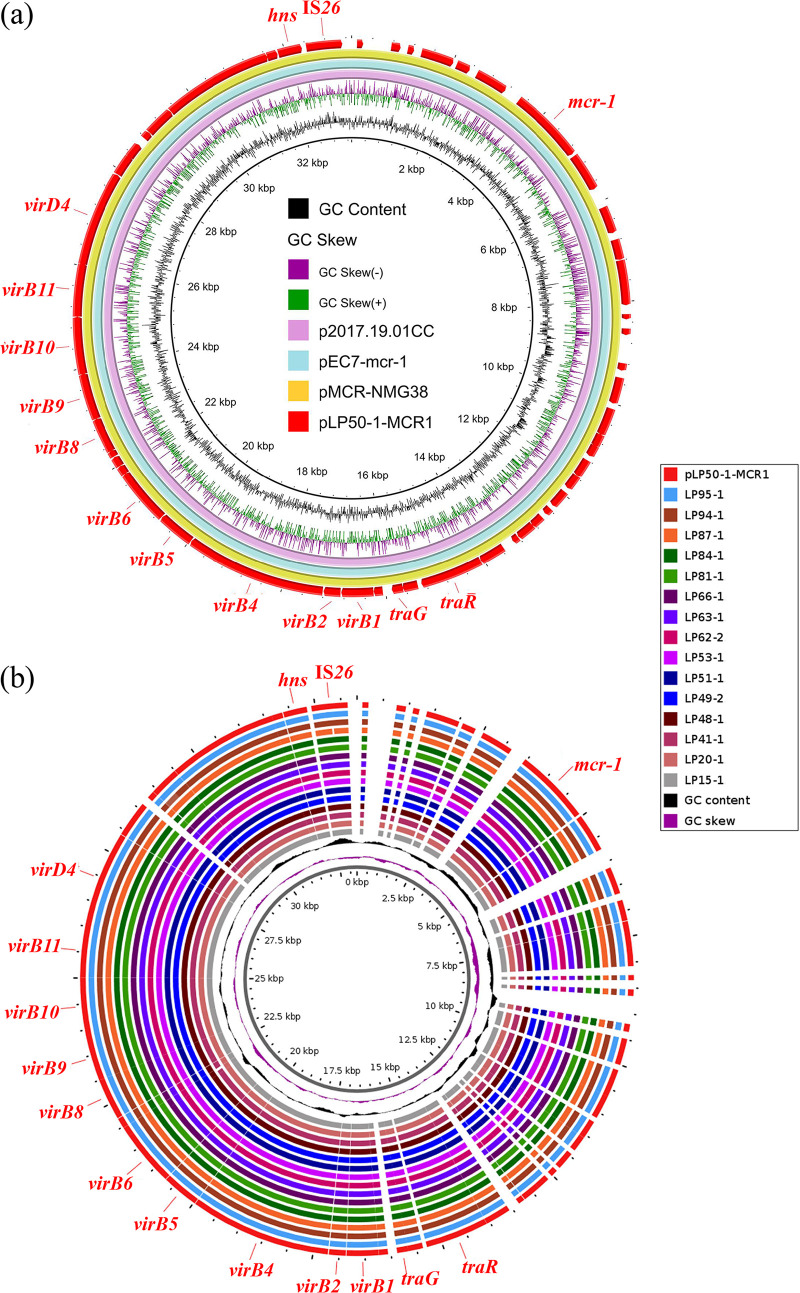
Circular comparison of *mcr-1*-bearing IncX4 plasmids. (a) Circular comparison of *mcr-1*-bearing plasmid pLP50-1-MCR1 with three similar IncX4 plasmids in the NCBI database. The outmost circle indicates the plasmid pLP50-1-MCR1 with genes annotated. (b) Circular comparisons between pLP50-1-MCR1 and draft assembly sequences of another 15 isolates carrying *mcr-1*-bearing IncX4 plasmids that have been sequenced with the Illumina HiSeq 2500 platform.

To investigate the genomic characterization of prevalent *mcr-1*-bearing IncX4 plasmids in the pigeon farm and the localization of *mcr-1* in LP5-1, isolates LP50-1 and LP5-1 were selected to be sequenced with the nanopore sequencing. Genetic analysis showed that isolate LP50-1 harbored a chromosome and seven plasmids consisting of pLP50-1-101kb, pLP50-1-87kb, pLP50-1-MCR1, pLP50-1-8kb, pLP50-1-4kb, pLP50-1-3kb, and pLP50-1-1kb. The *mcr-1* gene was located on the pLP50-1-MCR1 that was a typical IncX4-type plasmid. Sequence analysis revealed that the pLP50-1-MCR1 shared 99.99% identity at 100% coverage with plasmid p2017.19.01CC (LC511660) in E. coli isolated from humans in Vietnam and plasmid pEC7-*mcr-1* (CP060967) in E. coli isolated from humans in China. Additionally, it exhibited 99.98% identity at 100% coverage with plasmid pMCR-NMG38 (MK836307) in E. coli isolated from swine in China (Fig. 2a). These three similar plasmids are also *mcr-1*-positive plasmids. High similarity of these *mcr-1*-harboring IncX4 plasmids indicated the prevalence of E. coli carrying *mcr-1*-harboring IncX4 plasmids between animals and humans in different regions. Comparing pLP50-1-MCR1 and draft assembly sequences of another 15 isolates carrying *mcr-1*-bearing IncX4 plasmids that have been sequenced with Illumina HiSeq 2500 platform, we confirmed that all the *mcr-1*-harboring IncX4 plasmids have a similar structure (Fig. 2b). LP5-1 harbored a chromosome and six plasmids consisting of pLP5-1-106kb, pLP5-1-NDM-47kb, pLP5-1-37kb, pLP5-1-9kb, pLP5-1-3kb, and pLP5-1-1kb. The *mcr-1* gene was located on the chromosome and mediated by the IS*Apl1*-*mcr-1*-*pap2*-IS*Apl1*, which was called Tn*6330* mobile element and responsible for transfer of *mcr-1* (18). The pLP5-1-NDM-47kb was a typical IncX3 plasmid-carrying *bla*_NDM-5_. Isolates LP5-1, PT62, PT76, and PT77 had identical chromosomes and plasmids (Fig. 1a), but PT62, PT76, and PT77 additionally carried a transferable *tet*(X4)-bearing plasmid (16), indicating that the formation of PT62, PT76, and PT77 may be caused by horizontal transfer of *tet*(X4)-harboring plasmids.

### Characterization of nontransferable *mcr-1*-bearing IncX4 plasmids.

In total, the *mcr-1*-bearing IncX4 plasmids in 18 out of 39 isolates were nontransferable (Table 2). Six isolates carrying nontransferable IncX4 plasmids were sequenced with the Illumina HiSeq 2500 platform, and they belonged to ST646 (*n* = 5) and ST6164 (*n* = 1). However, nontransferable IncX4 plasmids had a highly similar structure to transferable IncX4 plasmids, including type IV secretion systems that are responsible for plasmid conjugative transfer. Therefore, we speculated that it may be factors other than the *mcr-1*-bearing IncX4 plasmids that cause this type of plasmid to fail to transfer horizontally. According to the results of PBRT, we found that all isolates carrying nontransferable IncX4 plasmids had the same replicons (IncX4, IncFIB, and IncF), except the ST6164 E. coli LP87-1. It is possible that all the isolates except LP87-1, carrying nontransferable IncX4 plasmids, were the ST646 E. coli (Fig. 1a and Table 2). This indicated that the ST646 E. coli isolates could spread *mcr-1*-bearing IncX4 plasmids by clonal dissemination rather than horizontal transfer. In fact, the phenomenon that commonly conjugative plasmids cannot be transferred into the recipient appeared in other studies as well (14, 19, 20).

### Fitness effects of *mcr-1*-bearing IncX4 plasmids.

**FIG 3:**
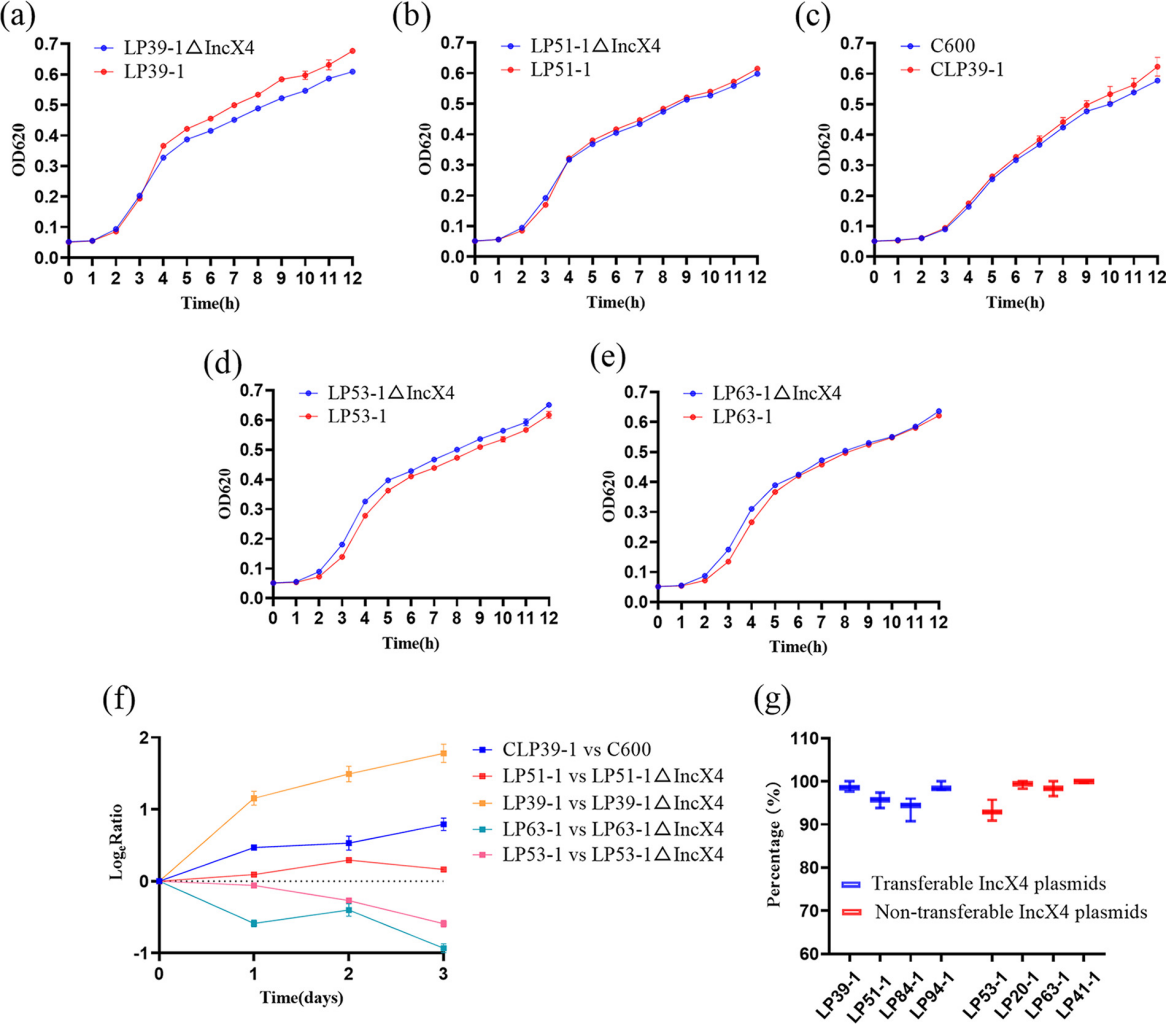
Fitness effects of *mcr-1*-bearing IncX4 plasmids on host. (a) Growth curves of LP39-1ΔIncX4 and LP39-1. (b) Growth curves of LP51-1ΔIncX4 and LP51-1. (c) Growth curves of C600 and CLP39-1. (d) Growth curves of LP53-1ΔIncX4 and LP53-1. (e) Growth curves of LP63-1ΔIncX4 and LP63-1. (f) Dynamics of competition experiments between plasmid-free E. coli isolates and E. coli isolates carrying *mcr-1*-harboring plasmids. (g) Stability of four transferable *mcr-1*-bearing IncX4 plasmids and four nontransferable *mcr-1*-bearing IncX4 plasmids.

Isolates LP53-1 and LP63-1 carrying nontransferable IncX4 plasmids and isolates LP39-1 and LP51-1 carrying transferable IncX4 plasmids were selected to study the fitness of IncX4 plasmids. We first acquired the *mcr-1*-positive transconjugant CLP39-1 of LP39-1, and eliminated *mcr-1*-bearing IncX4 plasmids by the CRISPR-Cas9 system in four isolates to acquire strains LP53-1ΔIncX4, LP63-1ΔIncX4, LP39-1ΔIncX4, and LP51-1ΔIncX4. Then we assessed plasmid fitness effects by measurement of growth curves and competition assays. Growth curves indicated that the growth rate of LP39-1ΔIncX4 was slower than that of LP39-1 (Fig. 3a). No significant differences in growth rates were observed between LP51-1 and LP51-1ΔIncX4 (Fig. 3b), and between C600 and CLP39-1 (Fig. 3c). However, the growth rate of LP53-1 was slower than that of LP53-1ΔIncX4 (Fig. 3d). The growth rate of LP63-1 was slower than that of LP53-1ΔIncX4 in the logarithmic growth phase, and there was no obvious difference between the two after the logarithmic growth phase (Fig. 3e). Competition assays showed that LP39-1, LP51-1, and CLP39-1 were more competitive than LP39-1ΔIncX4, LP51-1ΔIncX4, and C600, but LP53-1 and LP63-1 were less competitive than LP53-1ΔIncX4 and LP63-1ΔIncX4 (Fig. 3f). The above results suggested that carriage of transferable *mcr-1*-bearing IncX4 plasmids improves host fitness, but carriage of nontransferable *mcr-1*-bearing IncX4 plasmids imposed a burden on the host. A previous report indicated that the *mcr-1*-carrying IncX4 plasmid increased fitness of E. coli DH5α (19), which was consistent with our findings on strains in which *mcr-1*-carrying IncX4 plasmid was transferable. In addition, the transferability may contribute to increased fitness of IncX4 plasmids on host bacteria (21). The transferable *mcr-1*-bearing IncX4 plasmids make a significant contribution to the prevalence of *mcr-1* from the fitness perspective. We electroporated the nontransferable *mcr-1*-positive IncX4 plasmid of LP63-1 into LP39-1ΔIncX4 to acquire LP39-1ΔIncX4::IncX4, and found that the *mcr-1*-positive IncX4 plasmid could be transferred from LP39-1ΔIncX4::IncX4 into C600 again. We speculated that the transferability of these plasmids was related to genes of chromosome. Further study on the inhibitor to the transferability of IncX4 plasmids is required to contribute to the control of antimicrobial resistance. Eight isolates, including four isolates carrying transferable IncX4 plasmids and four isolates carrying nontransferable IncX4 plasmids, were randomly selected to assess stability of *mcr-1*-bearing IncX4 plasmids. The results showed that the transferable IncX4 plasmids and nontransferable IncX4 plasmids remained relatively stable after long-term passages (Fig. 3g). It is possible that the fitness advantage of transferable *mcr-1*-bearing IncX4 plasmids and the stability of *mcr-1*-bearing IncX4 plasmids allow *mcr-1* to remain prevalent without selection of colistin after colistin was banned as an animal feed additive.

### Conclusions.

To conclude, this study clearly illustrates that pigeons could act as reservoirs of *mcr-1*-positive E. coli in China. The prevalence of *mcr-1*-positive E. coli isolates from pigeons was mainly mediated by IncX4 plasmids, including transferable *mcr-1*-bearing IncX4 plasmids with fitness advantage in some E. coli isolates, and nontransferable *mcr-1*-bearing IncX4 plasmids with fitness disadvantage in some E. coli isolates. Plasmid transferability evaluation indicated that the same IncX4 plasmid has different transferability in different E. coli isolates. The transferability of these plasmids may be influenced by host chromosome in the same bacterial species. Additional research on the factors influencing the transferability of IncX4 plasmids in different bacterial hosts is required to help combat antimicrobial resistance. Continuous monitoring of *mcr-1*-positive E. coli from pigeons is necessary to understand its prevalence trends, and effective strategies to prevent such prevalence are urgently needed.

## MATERIALS AND METHODS

### Sample collection and identification of *mcr-1*.

In June 2021, 100 fresh fecal samples were collected from a pigeon farm located in Jiangsu, China. Samples were incubated in brain heart infusion (BHI) broth for 4 h at 37°C with continuous shaking (200 rpm). Then 100-μL cultures were transferred into fresh BHI broth containing colistin (2 mg/L) and incubated for 12 to 16 h at 37°C with shaking. Subsequently, colistin-resistant samples were plated on MacConkey agar plates and incubated for 12 to 16 h at 37°C. One or more colonies with varied morphological traits per sample were chosen and subsequently screened for the presence of *mcr-1* using PCR and Sanger sequencing (22). Bacterial species of all *mcr-1*-positive isolates were identified by 16S rRNA sequencing.

### Antimicrobial susceptibility testing.

Antimicrobial susceptibility profiles were determined for all the *mcr-1*-positive E. coli isolates by the broth microdilution method following the Clinical and Laboratory Standards Institute (CLSI) guidelines (23). We used the following 10 antimicrobial agents: doxycycline, enrofloxacin, ceftiofur, streptomycin, amoxicillin, rifampicin, florfenicol, meropenem, colistin, and tigecycline. Results were interpreted according to the CLSI standards (M100-S30 and M31-A3) and the European Committee on Antimicrobial Susceptibility Testing breakpoints (EUCAST, version 12.0) (http://www.eucast.org/clinical_breakpoints/).

### Conjugation experiments and plasmid replicon typing.

Conjugation experiments were done using *mcr-1*-bearing isolates as donors and E. coli C600 (rifampicin resistance) as the recipient strain. E. coli J53 (sodium azide resistance) was employed as the recipient if the isolate was resistant to rifampicin or failed to conjugate with E. coli C600. Briefly, the donor and recipient cultures were mixed in a 1:1 proportion, and 0.1 mL of the mixed cultures were plated onto solid lysogeny broth (LB) medium and incubated at 37°C overnight. Subsequently, conjugation mixtures on LB agar plates were collected and diluted in sterile saline. Transconjugants were selected by streaking the conjugation mix on LB agar plates supplemented with colistin (2 mg/L) and rifampicin (300 mg/L) or sodium azide (200 mg/L). The presence of *mcr-1* in transconjugants was checked by PCR and colistin resistance phenotypes. The PBRT (24) and the specific primers targeting the IncX4, IncHI2, and IncI2 replicons (13) were used to identify the plasmid replicons of *mcr*-positive isolates and corresponding transconjugants.

### Genome DNA sequencing and bioinformatic analysis.

According to MICs, transferability of *mcr-1*, and replicon types, 21 *mcr-1*-carrying isolates were selected to perform the genome sequencing. Genome DNA extractions were performed using the FastPure Bacteria DNA Isolation minikit (Vazyme, China) following the manufacturer’s instructions. The sequencing was conducted using the Illumina HiSeq 2500 platform to generate accurate short raw reads. SPAdes was used to assemble the short-read Illumina raw sequences to acquire draft genomes (25). The multilocus sequence typing (MLST), plasmid replicons, and antimicrobial resistance genes were analyzed using tools MLST (26), PlasmidFinder (27) and ResFinder (28) (https://www.genomicepidemiology.org/services/). Two representative isolates were further sent out for QitanTech nanopore single-molecule long-read sequencing (16, 29). To acquire the complete sequence of chromosome and plasmids, Unicycler was used to perform *de novo* assembly with the hybrid strategy based on the Illumina short-read data and QitanTech nanopore long-read data (30, 31). The complete genome sequences were then annotated using Rapid Annotation using the Subsystems Technology annotation website server (https://rast.nmpdr.org/rast.cgi) (32). The draft genomes were annotated using Prokka (33), and a pan-genome analysis was conducted using Roary (34). The phylogenetic tree was constructed using FastTree based on single nucleotide polymorphisms of core genomes (35) and visualized by iTOL (36). TBtools was used to visualize the distributions of antimicrobial resistance genes (37). Circular comparisons between *mcr-1*-harboring plasmids were performed using the BRIG (38). Comparisons between plasmids and draft genome sequences were performed using the website server (https://server.gview.ca/).

### Curing of *mcr-1*-bearing IncX4 plasmids.

Plasmid pCure-rif was employed to cure *mcr-1*-bearing IncX4 plasmids in E. coli. pCure-rif was constructed using the ClonExpress II One Step Cloning kit (Vazyme, China) as follows. First, we replaced the kanamycin resistance gene in pSGKP-km (Addgene plasmid ID: 117233) (39) with the rifampicin resistance gene *arr-2* from the clinical isolate PK8215 (CP080122) to generate plasmid pSGKP-rif. Then the gene encoding the Cas9 nuclease was amplified from plasmid pCasKP-hph (Addgene plasmid ID: 117232) (39) and cloned into pSGKp-rif, creating the plasmid pSGKp-Cas9-rif. Finally, the *araC* gene and l-arabiinducible promoter *araBAD* were amplified from pCasKP-hph and subsequently inserted into the plasmid pSGKp-Cas9-rif as the promoter for Cas9, resulting in pCure-rif. The 20-nt base-pairing region of an sgRNA targeting the *mcr-1* gene was designed through the online tool CHOPCHOP (http://chopchop.cbu.uib.no/). The paired oligonucleotides were randomly selected (*mcr*-spacer-F: tagtAAAGCTGTTTGATGTCACCG and *mcr*-spacer-R: aaacCGGTGACATCAAACAGCTTT), then annealed and ligated to the BsaI-digested pCure-rif using T4 DNA ligase (NEB, USA), resulting in pCure-rif-mcr, and then electroporated into competent cells of isolates carrying *mcr-1*-bearing IncX4 plasmids, followed by selection on agar plates supplemented with 100 mg/L rifampicin. Overnight cultures of strains carrying pCure-rif-mcr were diluted in 1 mL LB broth containing 0.1% l-arabinose in combination with 100 mg/L rifampicin and incubated for 12 h at 37°C. Then, the culture was plated on LB agar plates, and colonies lacking *mcr-1*-bearing IncX4 plasmid were selected and confirmed by PCR. For curing plasmid pCure-rif-mcr, colonies lacking *mcr-1*-bearing IncX4 plasmid were streaked onto LB agar plates containing 5% sucrose at 37°C for 12 h.

### Measurement of growth curves.

Overnight cultures from single colonies were adjusted to the 0.5 McFarland standard. Then, 5 μL adjusted cultures were diluted into 5 mL LB broth and incubated at 37°C, 200 rpm for 12 h. Bacterial growth was monitored by measuring the OD620 every 1 h using Multuskan FC (Thermo Fisher Scientific) (40).

### Competitive fitness.

The relative competitive fitness of plasmid-carrying clones was determined in pairwise serial competition experiments with the isogenic plasmid-free strain. Briefly, overnight cultures of each competitor were adjusted to the 0.5 McFarland standard and mixed in a 1:1 ratio, and 0.05 mL mixed competitors were transferred into 5 mL fresh LB broth (day 0). After 24 h of growth at 37°C, 0.05 mL cultures were transferred into 5 mL fresh LB broth (day 1), and then two transfers were performed (days 2 and 3). The colony-forming unit (CFU) of each competitor were determined by plating serial dilutions on antibiotic-free LB plates and selective plates containing 2 mg/L colistin. log_e_ ratio was calculated as follow: log_e_ ratio = log_e_ R(t)–log_e_ R(0), where “R” represents the ratio of the CFU of plasmid-bearing and plasmid-free cells in the competing cultures and “t” represents the time in days. If there is no difference in fitness between competing strains, the log_e_ ratio will be considered 0. If plasmid carriage improves host fitness relative to that of plasmid-free strains, log_e_ Ratio is positive, and it is negative if plasmid carriage reduces host fitness (40, 41).

### Plasmid stability.

To learn the stability of *mcr-1*-bearing IncX4 plasmids, amounts of 5 μL overnight cultures from single colonies were transferred into 5 mL antibiotic-free LB broth on the first day. Then, serial transfers of 5 μL cultures to 5 mL fresh LB broth were performed every 12 h and passaged for 30 days (60 passages; ~600 generations). Cultures from 60 passages were serially diluted in 0.9% saline and plated onto LB plates without antibiotics and LB plates supplemented with colistin (2 mg/L). The stability frequency was calculated as follow: (CFU on LB plate containing colistin/CFU on antibiotic-free LB plate) × 100% (40).

### Data availability statement.

The nucleotide sequences acquired in this study have been deposited under the NCBI BioProject with the accession number PRJNA861424.
